# Melanin-like nanoparticles as a potential novel therapeutic approach in ADPKD

**DOI:** 10.1038/s44321-024-00173-4

**Published:** 2024-11-20

**Authors:** Laura Cassina, Alessandra Boletta

**Affiliations:** Division of Genetics and Cell Biology, IRCCS San Raffaele Scientific Institute, Milan, Italy

**Keywords:** Genetics, Gene Therapy & Genetic Disease, Urogenital System

## Abstract

L. Cassina and A. Boletta discuss the study by Sun et al, in this issue of EMBO Mol Med, that describes a new therapeutic approach based on melanin-like nanoparticles for Autosomal Dominant Polycystic Kidney Disease.

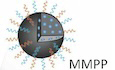

## Teaser

In this issue of *EMBO Molecular Medicine*, Sun and colleagues report the development of a novel therapeutic approach, and preclinical proof-of-concept evidence of its efficacy, for Autosomal Dominant Polycystic Kidney Disease (ADPKD) (Sun et al, 2024).

**Figure 1:**
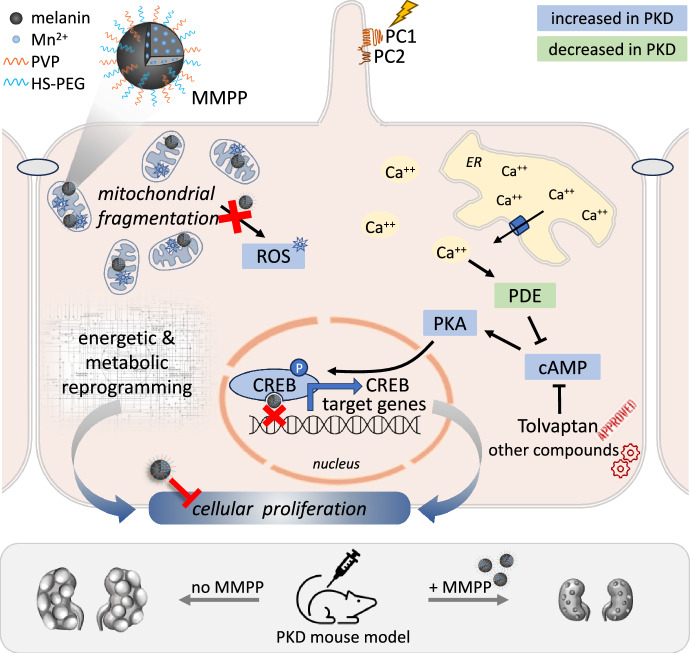
Schematic overview of the cellular effects of ultra-small melanin-like nanoparticles in PKD. Renal epithelial cells lacking PC1 expression show energy and metabolic rewiring, accompanied by mitochondrial fragmentation and increased reactive oxygen species (ROS) production. In parallel, aberrant cytoplasmic Ca^2+^ levels result in increased cAMP. High cAMP activates PKA, which in turn activates cyclic AMP-responsive element-binding protein (CREB). Therapeutic approaches targeting cAMP have been developed and resulted in improvement in disease progression, with Tolvaptan being the first approved treatment. A study in the current issue of *EMBO Mol Med* shows that ultra-small Mn^2+^-chelated melanin-like nanoparticles, incorporating polyvinyl pyrrolidone (PVP) and polyethylene glycol (PEG) (MMPPs, for short), exert dual activity: scavenging mitochondrial ROS, and inhibiting CREB transcriptional activity by preventing DNA binding. Based on this dual action, treatment with MMPPs delays the progression of cyst growth in the kidney and liver in a PKD mouse model.

ADPKD is one of the most prevalent monogenic disorders, characterized by bilateral renal cyst formation leading to progressive loss of kidney function. Furthermore, cysts are also frequently observed in the liver. While the precise molecular mechanisms driving cyst formation are not fully understood, several pathways contributing to cyst expansion have been identified (Zhou and Torres, 2022). Among these, elevated levels of cAMP are likely important in driving cell proliferation in the cystic epithelium (Zhou and Torres, 2022) (Fig. 1). Likewise, mitochondrial and metabolic dysfunctions are quite prominent, leading to an accumulation of reactive oxygen species (ROS) (Ishimoto et al, 2017; Padovano et al, 2018; Kahveci et al, 2020; Podrini et al, 2020; Cassina et al, 2020) (Fig. 1). To date, only one compound was developed and approved for the treatment of ADPKD: Tolvaptan, a Vasopressin type II receptor antagonist. This drug reduces cAMP production in the kidney’s collecting ducts, limiting cell proliferation and cyst expansion, as seen in preclinical models and human samples (Zhou and Torres, 2022) (Fig. 1).

However, Tolvaptan has limited effectiveness, is poorly tolerated, and can cause significant side effects, including rare but serious liver toxicity (Zhou and Torres, 2022). Therefore, there is a pressing need to develop new therapeutic options for ADPKD.

Sun et al develop a novel approach based on melanin-like nanoparticles to improve disease progression in animal models of PKD, and attribute its efficacy to a dual mechanism of inhibition of cAMP/CREB activation and of ROS production (Sun et al, 2024) (Fig. 1).

Melanin-like nanoparticles (MNPs) are effective agents in antioxidant therapy (Sun et al, 2019), due to their strong ability to neutralize reactive oxygen species (ROS), and the authors set out to test whether this could be a valuable approach to halt disease progression in PKD. They prepared and analyzed the biodistribution of ultra-small MMPP, showing that they can cross the glomerular filtration barrier and be re-absorbed by proximal tubules and collecting ducts.

The newly prepared MMPP formulation was used in vitro on MDCK cells grown suspended in 3D gels following forskolin treatment, or ex vivo on a renal cyst model of embryonic kidneys cultures supplemented with 8-Br-cAMP and in both models induced a reduction in the cystic burden.

Next, MMPP were tested in vivo in a mouse model carrying a ubiquitous inducible Cre line in combination with *Pkd1* floxable alleles, in which inactivation at P25-P28 resulted in a slowly progressive model of PKD, which allowed treatment and follow-up for three months (Sun et al, 2024). In line with the in vivo distribution of MMPP, both the kidney and the liver phenotypes were improved. MMPP treatment reduced kidney size and the kidney weight/body weight (KW/BW) ratio, and improved histological appearance, cyst area, and cystic index. Importantly, the renal function of PKD mice, measured by blood urea nitrogen and creatinine, was also significantly improved following MMPP treatment.

The authors then conducted transcriptomic analysis on collecting duct epithelial cells isolated from control, PKD, and MMPP-treated PKD mice. Distinctive patterns of gene expression were identified and classified in various clusters based on their response to disease and/or treatment. At least two clusters showed prominent changes in PKD kidneys that were effectively restored by MMPP treatment.

Among these clusters were mitochondrial genes, downregulated in PKD in line with the reported alterations in the morphology and metabolic function of mitochondria in PKD (Ishimoto et al, 2017; Padovano et al, 2018; Kahveci et al, 2020; Podrini et al, 2020; Cassina et al, 2020). The investigators also used primary renal tubule cells to measure oxygen consumption rate (OCR) and showed a rescue in the reduced OCR observed in the mutants. Notably, several of these beneficial effects were driven by a rescue in ROS accumulation through the upregulation of crucial genes involved in maintaining oxidative balance and ROS homeostasis.

The transcriptomic analysis also revealed that nearly one-third of the MMPP-rescued genes contained cAMP-responsive elements (CRE) sequences in their promoter and/or enhancer regions. Given the known activation of CRE-binding protein (CREB) in PKD kidneys, likely driven by the cAMP accumulation, and its role in promoting cyst growth in ADPKD (Zhou and Torres, 2022; Hansen et al, 2022), the authors tested the hypothesis that MMPP were acting directly by regulating this cascade.

Using Forskolin to induce cAMP, the investigators excluded that MMPP were modulating protein kinase A (PKA) activity or CREB phosphorylation (Fig. 1). In contrast, MMPP directly interacted with CREB, via one of its specific domains, the bZIP domain. Consistently, recombinant CREB proteins lacking the bZIP domain showed no interaction with MMPP. Similarly, purified mCherry-CREB ΔbZIP proteins failed to recruit MMPP into droplets, showing the importance of the CREB bZIP domain for direct interaction (Sun et al, 2024). Finally, the investigators provide evidence that MMPP, by interacting with CREB, impairs its ability to interact with the CRE sequences on genomic DNA.

Importantly, MMPP inhibition of CREB transcriptional activity was not secondary to ROS regulation, since treatment with sulforaphane (SFN) or *N*-acetylcysteine (NAC), two well-known antioxidants, rescued ROS levels, but did not impact CREB transcriptional activity. Thus, the strong efficacy observed both in the kidney and in the liver of cystic PKD mice is likely due to the dual action of MMPP, able to target two distinct, not interdependent, and equally important processes de-regulated in PKD: mitochondrial ROS accumulation and CREB abnormal transcriptional activity (Sun et al, 2024) (Fig. 1).

The study has multiple strengths. First, the development of novel, particularly small particles allows their use in various kidney diseases in which different renal nephron segments are affected. Second, the experiments in *Pkd1* mutant mice show a robust amelioration of both the kidney and the liver phenotype, which is particularly relevant since Tolvaptan does not provide benefit to liver cysts. The dual inhibitory effect of melanin-nanoparticles both on ROS production as previously reported (Liu et al, 2020), and on CREB increased transcription, might explain why this treatment is effective in reducing kidney and liver cysts and, most importantly, in improving renal function. The study also has some limitations for potential MMPP development into a clinical program. Melanin-nanoparticles have so far only been used in acute settings, such as acute kidney injury (Sun et al, 2019). PKD is a chronic disease, and safety issues linked to chronic treatment will need to be considered carefully. In particular, the study by Sun et al shows that several transcriptional alterations detected in PKD kidneys are not rescued by MMPP. Furthermore, several genes that appeared to be unaffected in *Pkd1* kidneys were severely affected by MMPP treatment. The transcriptional profiles of control kidneys treated with the same regimen were not provided and it is therefore unclear if these changes are specific to PKD kidneys. Transcriptional changes caused in mutant kidneys by any given drug would cause serious concerns of undesired toxicity, an issue particularly relevant for ADPKD, which is considered a relatively benign condition.

In conclusion, this study presents evidence of a very effective improvement of kidney and liver disease in a mouse model of PKD by using melanin-nanoparticles. The mechanism of action of the drug is interesting because it targets at least two key branches of PKD cystic epithelia dysfunction (i.e., ROS accumulation and CREB excessive transcriptional activity, Fig. 1). These findings expand the landscape of possible interventions for this orphan disease.
